# Impacts of Oxygen Tension on Developmental Competence of Preimplantation Embryos

**DOI:** 10.3390/biom16030341

**Published:** 2026-02-24

**Authors:** Shayesteh Mehdinejadiani, Brad Link, John P. Kastelic, Jacob Thundathil

**Affiliations:** 1 Faculty of Veterinary Medicine, University of Calgary, Calgary, AB T2N 1N4, Canada; shayesteh.mehdinejad@ucalgary.ca (S.M.); jpkastel@ucalgary.ca (J.P.K.); 2 Regional Fertility Program, 2000 Veterans Pl NW #400, Calgary, AB T3B 4N2, Canada; blink@regionalfertilityprogram.ca

**Keywords:** oxygen tension, in vitro fertilization, in vitro maturation, embryo culture, preimplantation development, reproduction, gene expression, epigenetic regulation

## Abstract

Oxygen (O_2_) tension is a critical factor influencing in vitro development of pre-implantation embryos. The in vivo environment has lower O_2_ tension (2–10%) than atmospheric air (~20%), along the female reproductive tract, from the oviducts (8–10%) to the uterus (2–5%), supporting development of early-stage embryos. As the female reproductive tract is inherently hypoxic, replicating low-O_2_ conditions in vitro may enhance embryo development. In contrast, culturing embryos under non-physiological O_2_ tension may impair stress adaptation and reduce developmental competence. Optimal O_2_ tension likely varies with species and embryo stage, suggesting a single uniform O_2_ tension throughout in vitro culture may not be ideal; conditions beneficial at one stage may be detrimental at another. Although atmospheric O_2_ harms embryo development and redox balance, specific advantages of low (5%) or ultra-low (≤2%) O_2_ remain uncertain, despite many studies documenting improved development under hypoxia. This review examines the current literature on effects of atmospheric, low, and ultra-low O_2_ tension during in vitro embryo culture, emphasizing impacts on in vitro fertilization (IVF) outcomes, and the regulation of transcription and epigenomics during pre-implantation embryo development.

## 1. Introduction

Assisted reproductive technologies (ARTs) have substantially advanced; however, their efficiency remains suboptimal. In vitro embryo production (IVEP) is a fundamental reproductive biotechnology with widespread applications in animal and human reproduction and research. Optimizing culture systems is crucial for improving IVEP success rates and reducing numbers of cycles required to achieve healthy offspring.

Various environmental factors, including non-physiological pH, temperature, and oxygen (O_2_) tension, can negatively impact the efficiency of embryo culture [1,2,3]. The role of O_2_ in cellular respiration and energy production underscores its importance, as deviations from physiological O_2_ tension may adversely affect embryo viability and developmental potential [4]. For years, embryo culture was routinely performed under atmospheric O_2_ tension (~20%) [5,6]. The importance of O_2_ tension in embryo culture was initially highlighted in the earliest successful report of human embryo culture to the blastocyst stage, which used a low O_2_ environment of 5%, suggesting early recognition of the importance of mimicking in vivo conditions [7]. Since then, numerous studies have investigated impacts of O_2_ tension on pre-implantation embryo development in vitro [8,9,10,11,12,13]. As the embryo moves from the oviduct to the uterus, it is exposed to increasingly hypoxic conditions, a change that aligns with a metabolic shift from oxidative phosphorylation prior to compaction to glycolysis at the morula stage [14]. These coordinated metabolic and morphological changes indicate that a static O_2_ environment may not support optimal embryonic development in vitro.

Optimal O_2_ tension for embryo culture may vary depending on the stage of embryonic development [4]. Steptoe et al. [7] utilized a tri-gas system (5% CO_2_, 5% O_2_, 90% N_2_) for the first successful in vitro culture of human embryos to the blastocyst stage. Subsequent studies [14,15] demonstrated detrimental impacts of atmospheric O_2_ tension on embryo development. Most studies compared atmospheric O_2_ to low O_2_ (5%) [16,17,18]. Exposure to atmospheric O_2_ significantly impacted embryo development, leading to alterations in developmental kinetics, transcription [19,20,21,22], histone remodeling and methylation patterns [23,24,25], as well as changes in the proteome [26] and metabolic state [27,28,29]. Several studies suggested that the benefits are greatest when embryos are cultured under low O_2_ tension throughout the entire culture period, from Day 0 to Days 5 or 6 [30,31,32].

Although 5% O_2_ culture systems are increasingly being adopted, there is growing interest in sub-5% O_2_ systems [4,33,34,35], driven by evidence that uterine O_2_ tension in primates and human ranges from 1.9 to 2.6% [36]. Most body tissues, including the reproductive system, operate optimally at O_2_ tension ranging from 2 to 10% [3], suggesting that atmospheric O_2_ may have detrimental effects on fetal development and long-term health of the offspring. Since O_2_ tension is controllable, understanding its impacts on embryo culture will inform strategies for refining embryo culture conditions, enhancing embryo development and clinical outcomes.

## 2. Oxygen Tension in the Female Reproductive Tract

Several studies focused on defining O_2_ tension within the female reproductive tract, aiming to understand its role in embryo development and implantation. There are significant interspecies and intraspecies differences in O_2_ tension within intratubal and intrauterine environments [35]. Early investigations, dating back to the 1950s, analyzed luminal fluids of the mammalian oviduct and reported that O_2_ tension typically ranges from 2 to 8% [37,38].

During initial cleavage stages in vivo, from Days 1 to 3, an embryo is exposed to an O_2_ tension ranging from 5 to 7% in several mammals, including rhesus monkeys, hamsters, and rabbits. However, as the embryo enters the uterine cavity late Day 3, advancing from the 8-cell stage to a morula and eventually forming a blastocyst, O_2_ tension gradually declines to ~2% at implantation [35]. The uterus is characterized by a persistently hypoxic environment throughout the first trimester of pregnancy [39], crucial in supporting vasculogenesis and promoting rapid cellular proliferation, akin to mechanisms observed in tumor growth [40]. This hypoxia may be a physiological mechanism to protect the developing embryo from potential O_2_-induced toxicity [3].

Massa et al. [38] proposed that, in the absence of ovulation, O_2_ supplied by blood to the oviduct is only adequate to meet the tissue’s basic requirements. However, ovulation triggers a modest increase in O_2_ delivery for the next 5–7 days, specifically in the ipsilateral oviduct, supporting gamete survival, fertilization, and early embryonic growth. Perhaps oocyte–cumulus complexes (COCs) are a stimulus, enhancing local blood flow enough to elevate O_2_ tension in tubal fluid, thereby supporting these critical reproductive processes.

In 1993, Fischer and Bavister conducted a pivotal study that advanced understanding of reproductive O_2_ environments in vivo across several species, including rhesus monkeys, rabbits, and hamsters. Their research provided the first measurements of intrauterine O_2_ tension in primates during pregnancy and pseudopregnancy. Notably, O_2_ tension within the uterus and oviduct were considerably lower than atmospheric tension. Oviductal and uterine O_2_ tension in rhesus monkeys were ~35–71 mmHg (4.9–10%) and 11–14 mm Hg (1.5–2% O_2_), respectively [35]. In rabbits, pooled mean O_2_ tension was significantly higher in the oviduct (~53 mmHg; 7.4% O_2_), compared to the uterus (~24 mmHg; 3.5% O_2_). However, in hamsters, there was no significant difference in O_2_ tension between the oviduct (~45 mmHg; 6.3% O_2_) and the uterus (~37 mmHg; 5.3% O_2_) [35].

Ottosen et al. measured intrauterine O_2_ tension in humans using a fiber-optic microsensor with a 0.9 mm diameter, recording values every 5–10 min. Average O_2_ tension was ~18.9 mmHg, equivalent to 2.5–2.6% O_2_ tension [36]. O_2_ is essential for maintaining both sperm motility and fertility. Consequently, it is physiologically relevant that intraluminal O_2_ tension during insemination is elevated compared to the minimal tension required to sustain sperm viability [41]. As outlined above, O_2_ tension in the reproductive tract is considerably lower than atmospheric tension and, in some species, varies depending on the specific organ and phase of the ovarian cycle. Therefore, characterizing O_2_ tension in vivo is crucial for developing effective in vitro embryo culture systems.

## 3. Oxygen Tension in Culture Systems and Its Effects on Embryo Development: From Past to Present

Cyclic and minute-to-minute fluctuations in O_2_ tension occur in the female reproductive tract. These dynamic changes are influenced by various factors, including uterine contractility, autonomic nervous system activity, hormonal variations, integrity of myometrial and smooth muscle tissues, and cardiac pulsatility [3]. Although it may not be possible to control some of these variables in an in vitro culture system, it is prudent to focus on elements that can be controlled, such as composition of culture medium, temperature, pH, and O_2_ tension, especially considering that in culture media equilibrated with atmospheric O_2_ (178 µmol/L), O_2_ tension is substantially higher than physiological intracellular O_2_ tension (10 µmol/L) [42].

While physiological oxygen concentrations measured within the female reproductive tract provide an important biological reference point, physiological relevance should not be automatically equated with functional optimality in an in vitro culture system. In vivo, the pre-implantation embryo develops within a highly regulated and dynamic microenvironment supported by maternal antioxidant defenses, metabolic buffering systems, paracrine signaling, vascular perfusion, and spatial–temporal oxygen gradients. In contrast, in vitro culture represents a comparatively static and simplified environment in which these buffering and adaptive mechanisms are absent or substantially reduced. Under such conditions, embryonic responses to reduced oxygen tension may differ fundamentally from those occurring in vivo. Therefore, optimization of O_2_ tension in culture systems should be guided not solely by attempts to replicate physiological measurements, but by careful evaluation of developmental competence, genomic stability, and long-term safety within the specific constraints of the in vitro setting.

### 3.1. Impacts of Oxygen Tension on In Vitro Maturation (IVM) and Oocyte Competence

**Figure 1:**
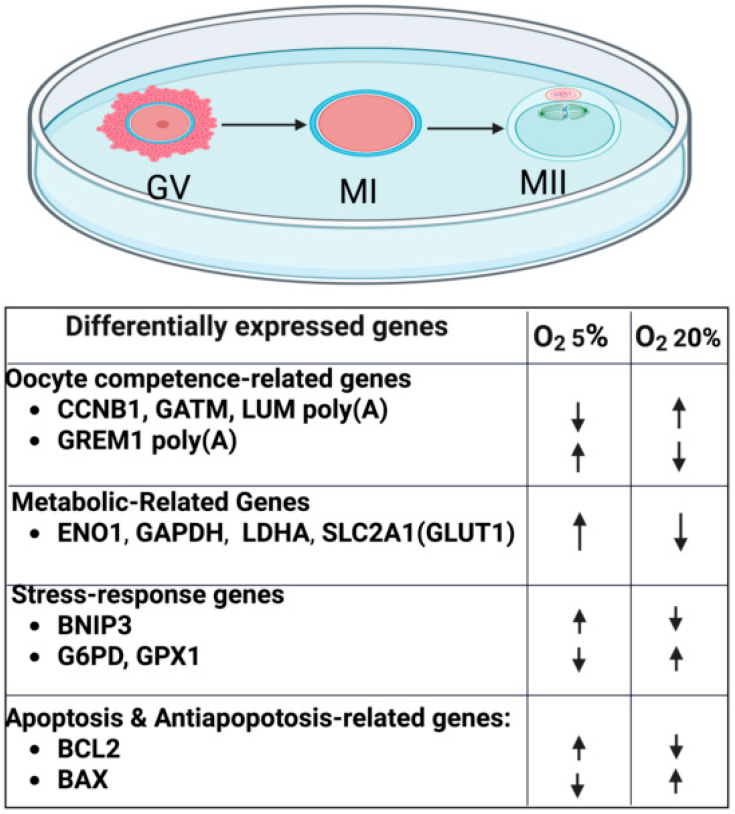
Effects of low (5%) O_2_ vs. atmospheric (20%) O_2_ on transcriptional alterations during in vitro maturation of oocytes. The direction of the arrow heads indicates up- or downregulation of gene transcription. GV: Germinal Vesicle, MI: Metaphase I, MII: Metaphase II, CCNB1: Cyclin B, GATM: Glycine Amidinotransferase (L-arginine:glycine amidinotransferase), LUM: Lumican, GREM1: Gremlin 1, DAN Family BMP Antagonist, ENO1: Enolase 1, GAPDH: Glyceraldehyde-3-phosphate dehydrogenase, LDHA: Lactate Dehydrogenase A, SLC2A1: Solute Carrier Family 2 Member 1, GLUT1: Glucose Transporter 1, BNIP3: BCL2 Interacting Protein 3, G6PD: Glucose-6 phosphate dehydrogenase, GPX1: Glutathione peroxidase 1, BCL2: B-cell lymphoma 2, BAX: BCL2-associated X protein. Created in BioRender. Mehdinejadiani, SH. (2026) https://BioRender.com/7qm2flx.

Ovarian follicles develop in a naturally hypoxic, avascular environment, where hormones like LH and FSH regulate folliculogenesis, oocyte maturation, and ovulation. This hypoxia activates hypoxia-inducible factors (HIFs) that regulate genes such as VEGF, a key regulator of angiogenesis. Although the role of HIFs in ovulation is well-established, their involvement in oocyte maturation remains unclear, partly due to the difficulty of replicating in vivo O_2_ tension in vitro [43]. Most IVM protocols use ~20% O_2_, far exceeding the physiological range in follicles, typically 3 to 13%, depending on species [44]. In human follicular fluid, dissolved O_2_ tension ranges from ~1.5 to 6.7%, underscoring the gap between in vitro and in vivo O_2_ conditions [45] to which oocytes are exposed. Low O_2_ tension (5% O_2_) during IVM enhanced developmental competence of oocytes across species [cattle [46,47,48,49], buffalo [50,51], pigs [52,53], and mice [54], including improved expression of activation markers (Figure 1), higher-quality blastocysts, and favorable transcriptomic changes, although fertilization or maturation rates were unchanged (Table 1).

Standard IVM induces premature nuclear maturation without cytoplasmic readiness due to loss of follicular regulation. A novel biphasic IVM system, termed capacitation IVM (CAPA-IVM), was developed by Sanchez et al. using human oocytes retrieved from small follicles of patients with polycystic ovary syndrome [64] synchronizing cytoplasmic and nuclear maturation of oocytes, improving developmental competence and resulting in live births [65,66,67]. In CAPA-IVM, C-type natriuretic peptide (CNP), a physiological follicular factor, is added during the pre-maturation phase to maintain meiotic arrest and prevent premature oocyte maturation [64,65]. Using a biphasic strategy that includes pre-maturation culture (PMC) with CNP (24 h) under 20% O_2_ tension, followed by IVM supplemented with FSH and amphiregulin (30 h), enhanced oocyte maturation capacity by synchronizing cytoplasmic and nuclear maturation, improving developmental competence, with more Day-3 embryos, high-quality blastocysts suitable for single-embryo transfer, and successful live births [64]. Recently, a human study further demonstrated that culturing cumulus–oocyte complexes under low O_2_ (5%) during the pre-IVM phase significantly improved metabolic efficiency. Despite reduced mitochondrial respiration, COCs exhibited higher ATP content, stable glucose uptake, and increased lactate production, indicating a metabolic shift toward more efficient energy utilization, potentially involving alternative substrates such as fatty acids. Importantly, mitochondrial membrane potential and antioxidative capacity remained unchanged, supporting the notion that low O_2_ better preserves cellular homeostasis during biphasic IVM. These findings emphasized the importance of optimizing O_2_ tension in pre-IVM culture to more closely mimic in vivo follicular conditions and improve human oocyte competence [62]. In contrast, applying 5% O_2_ throughout both CAPA-IVM phases led to fewer mature oocytes and lower fertilization (2PN) rates in patients with polycystic ovary syndrome [63].

Regarding complementary evidence from controlled animal models, in a mouse study, reduced O_2_ tension during the pre-IVM phase was examined using a biphasic CAPA-IVM system. There were no significant differences between 5% and 20% O_2_ groups in oocyte maturation, oocyte diameter, cleavage, or blastocyst rates. However, COCs exposed to 5% O_2_ during the CAPA step had mitochondrial respiratory profiles closer to in vivo-matured COCs [55]. Building on these observations, O_2_ tension markedly influenced oocyte metabolic regulation during IVM of porcine oocytes. Continuous low oxygen (5% O_2_) did not affect blastocyst rates but enhanced oocyte ATP and lipid content, reduced mitochondrial ROS and membrane potential, increased glucose uptake in COCs, and decreased mitochondrial DNA and protein content in cumulus cells. Transcriptomic profiling confirmed a metabolic shift toward glycolysis with suppression of oxidative phosphorylation and steroidogenesis pathways under low O_2_. Moreover, a biphasic oxygen protocol further improved oocyte metabolic status and embryonic developmental competence. Collectively, these findings indicated that early low-O_2_ exposure promoted porcine oocyte quality primarily through the modulation of energy metabolism [57].

Similar to observations in other species, the interaction between O_2_ tension and glucose concentration also influences bovine oocyte maturation outcomes. Low O_2_ (5%) in the presence of high glucose (20 mmol/L) was associated with improved developmental competence [47], possibly by promoting anaerobic glycolysis in COCs. This may enhance oocyte competence through increased energy production and reductions in ROS and mitochondrial damage [46]. However, oocytes are highly sensitive to glucose concentrations; as either deficient (<2.3 mM) or excessive (>10 mM) glucose can impair nuclear and cytoplasmic maturation and compromise embryo development [68]. Reduced O_2_ has been proposed to better mimic physiological conditions, but results across species remain inconsistent. Therefore, further studies on interactions between O_2_ tension and glucose concentration are critical for creating an optimal microenvironment for oocyte maturation and its developmental competence.

### 3.2. Reduced Oxygen, Hypoxia (Low) and Ultra-Hypoxia (Ultra-Low) in Embryo Culture Systems

Early studies examining impacts of O_2_ tension on embryo development produced inconsistent findings, with no significant differences in fertilization, cleavage, pregnancy, or implantation rates when embryos were cultured up to Days 2 or 3 (with Day 0 defined as the day of fertilization) under low-O_2_ versus atmospheric O_2_ conditions [69,70,71,72]. However, more recent evidence demonstrated that physiological O_2_ tension improves both laboratory parameters and clinical outcomes, suggesting a growing consensus on the benefits of reduced O_2_ tension during embryo culture [4,17,27,73,74,75,76].

Based on animal studies, culturing embryos under low O_2_ tension (5% O_2_) consistently improves developmental outcomes across species, including mice [77], cattle [78,79], and buffalo [80]. Future evidence indicates that embryos exposed to atmospheric O_2_ (20%) had lower blastocyst hatching rates, reduced total cell numbers, and diminished embryo quality compared to those cultured under 5% O_2_. Notably, buffalo embryos developed under 5% O_2_ also had improved post-thaw survival, highlighting the broader advantages of low-O_2_ culture across mammals [80]. At the molecular level, further assessments confirmed better outcomes for both the IVM procedure [61] and embryos cultured at 5% O_2_ compared to those maintained under 20% O_2_ or ultra-low-O_2_ conditions (1% O_2_) [61,80].

To extend these observations beyond experimental models, clinical studies in humans have reported that, although varying O_2_ tension had no significant impact on fertilization rates, blastocyst formation and quality, or pregnancy outcomes, embryos cultured under low O_2_ tension had a significantly higher mean embryo score on Day 3 [81]. With advancements in culture media, embryos derived from sibling oocytes cultured under 5% O_2_ tension throughout the culture period had significant improvements in total blastocyst yield, as well as in quality of Day-3 and Day-5 embryos [32,82]. Improved blastocyst development under low O_2_ tension has been associated with significantly higher pregnancy and live birth rates [18,30,31,76,83,84,85].

Based on time-lapse imaging, pre-implantation embryos from both humans and mice have delayed development when cultured under atmospheric O_2_ conditions, in contrast to those maintained in a low-O_2_ environment [27,28]. However, Santos et al. suggested that low O_2_ tension during embryo culture does not significantly impact global metabolism of human cleavage-stage embryos [86]. Although low O_2_ tension (e.g., 6% O_2_) has been associated with improved embryo quality, randomized clinical trials have consistently shown no significant differences in ongoing pregnancy rates or live birth rates per transfer when comparing embryos cultured under low (6%) versus atmospheric (~20%) O_2_ tension [69,83]. Nevertheless, in recent years, the standard practice in many ART laboratories has shifted toward culturing embryos under 5% O_2_ using tri-gas incubator systems, rather than at an atmospheric tension of 20% O_2_ [87]. This approach is also endorsed by the European Society for Human Reproduction and Embryology (ESHRE) in their 2015 updated guidelines for best practices in in vitro fertilization (IVF) laboratories (Table 2) [88].

Notably, findings of even lower O_2_ tension in the uterus compared to the oviduct, such as 1.5–2.0% in monkeys, 5.3% in hamsters, and 3.5% in rabbits, have prompted several research groups to advocate for embryo culture under 2% O_2_ to better mimic the in vivo environment [4,33,34,35,92,97]. Nakagawa et al. reported that culturing human embryos under ultra-low O_2_ tension (2%) did not significantly improve ART outcomes compared to those cultured at 5% O_2_. Therefore, although lower O_2_ tension may negatively impact embryo morphology, they do not appear to compromise embryo viability [97]. This study served as a valuable starting point for exploring effects of O_2_ tension below 5%; however, culturing embryos in a 2% environment has produced inconsistent outcomes across various studies. Interestingly, continuous culture of human embryos from Day 0 to Days 5 or 6 under 3.5% O_2_ conditions significantly reduced blastocyst formation, with poorer clinical outcomes, despite higher fertilization and cleavage rates [98].

To further clarify whether these observations are species-specific or represent a conserved biological response, several animal studies have investigated ultra-low-O_2_ conditions. Mouse embryos, cultured under 2% O_2_, resulted in decreased blastocyst yield, lower average cell number per blastocyst, reduced blastocoel cavity size, and diminished expansion compared to embryos cultured under 5% O_2_ [92]. This may be attributed to prolonged hypoxic culture impairing cellular proliferation, consistent with human embryonic stem cells, where prolonged exposure to ultra-low O_2_ (1.5%) during differentiation was associated with reduced proliferation and increased apoptosis [107]. Similarly, time-lapse morphokinetic analysis revealed no observable advantage of culturing mouse embryos under 2% versus 6% O_2_ [91]. In yak oocytes and blastocyst cells, there was a significant reduction in apoptotic cell numbers under 5% O_2_ tension; however, there was a notable increase in the 1% O_2_ tension group [61]. Perhaps both atmospheric O_2_ and ultra-low-O_2_ conditions contribute to the induction of apoptosis (Table 2).

### 3.3. Biphasic Oxygen Strategies in Culture Systems: A New Frontier for Enhancing Embryo Competence and Clinical Success

Distinct developmental stages likely require different O_2_ tension, as an O_2_ tension that supports embryo development at one stage may have adverse effects at another. This highlights the importance of tailoring O_2_ conditions to the specific needs of each developmental phase to optimize embryo viability and progression in humans [33]. Findings on uterine O_2_ tension, together with the observation that embryos reach the uterus around the time of compaction (approximately late Day 3), suggested that mimicking a natural O_2_ gradient, rather than a constant 5% O_2_ tension, could be more physiologic for embryo culture [27,34,108]. Although several human studies have reported no significant advantage in embryo development when using biphasic culture systems that lowered O_2_ tension from 20 to 5% [28] or from 5 to 2% O_2_ [14], compared to monophasic hypoxic culture conditions, O_2_ tension can influence embryonic development at both cleavage and post-compaction stages [27,109].

Yang et al. compared the extended culture of thawed human embryos under three O_2_ tensions (2, 5 and 20%). These embryos had been previously cultured up to Day 3 in 20% O_2_ and cryopreserved, and the study evaluated their subsequent development to the blastocyst stage under varying O_2_ tension. Culturing thawed embryos to the blastocyst stage under 5% O_2_ tension resulted in optimal developmental outcomes; they had improved growth, reduced apoptosis, and less oxidative stress compared to those cultured under 2 or 20% O_2_ tensions [33]. However, exposure to 20% O_2_ during culture up to Day 3 prior to cryopreservation may have confounded assessment of effects of 2% O_2_ culture on development.

Previous studies explored whether culturing human embryos under O_2_ tensions lower than the 5%, either entirely or partially, could more closely mimic physiological conditions. Human zygotes were cultured under three conditions: 2 or 5% O_2_, or a sequential gradient of 5% for Days 1–3 to 2% O_2_ until Days 5/6. The group exposed to the 5 to 2% O_2_ gradient had remarkably improved embryo development and favorable morphological grading relative to the 5% O_2_ group, without the increased apoptosis observed in the 2% O_2_ group [110]. Perhaps sequential O_2_ tension in a culture system may better replicate in vivo conditions than a constant 5% O_2_ environment, with potential for improving human embryo culture outcomes [34,99,106,110]. Notably, clinical evidence from human IVF cycles indicates that the biphasic O_2_ strategy (5 to 2%) significantly enhanced the number of usable blastocysts and cumulative live birth rates [99,106]. This biphasic culture approach may be a valuable alternative for increasing production of euploid, developmentally competent blastocysts, particularly in patients with few blastocysts or poor prognosis [103,104]. Mechanistically, the improvement in euploid blastocyst formation under biphasic O_2_ (5 to 2%) conditions is likely driven by reduced oxidative stress during critical stages of chromosome segregation. Animal and experimental data suggest that lower ROS concentrations may preserve spindle integrity, kinetochore–microtubule attachments, and DNA repair capacity, whereas the ultra-low O_2_ phase (2%) during blastocyst formation may further facilitate embryonic self-correction through selective elimination of chromosomally abnormal blastomeres, collectively increasing the likelihood of euploid blastocyst development [103]. However, the extent to which these mechanistic processes observed in animal models occur in human embryos remains to be fully determined [111,112,113,114].

Although human data support the benefit of biphasic O_2_ strategies, animal studies provide important mechanistic insights into how O_2_ tension differentially regulates embryo development in species- and stage-dependent manners. In 2003, Yuan et al. conducted a study using a bovine model to examine the effects of O_2_ tension (2, 5 or 20%, and a sequential reduction from 20 to 5% after 72 h) on cell number and apoptosis. Blastocysts cultured under 5% O_2_ and the sequential 20-to-5% O_2_ conditions had significantly higher total cell counts compared to those cultured in 2 or 20% O_2_. Additionally, these groups had a reduced incidence of apoptosis, significantly lower than in the 20% O_2_ group, though not significantly different from the 2% group. However, hatching rate was lower in the sequential 20 to 5% group compared to the 5% O_2_ group. ROS, particularly at the blastocyst stage, have been linked to increased apoptosis, which may account for the higher incidence of cell death in embryos cultured under 20% O_2_. Apoptotic cells were detected in both the inner cell mass (ICM) and the trophectoderm, with a higher incidence of apoptosis in the ICM [96].

In a recent study investigating bovine embryo development, embryos were cultured under three O_2_ conditions: atmospheric O_2_ (20%), low O_2_ (6%), and a biphasic low/ultra-low regimen, with embryos exposed to 6% O_2_ until the 16-cell stage and then transitioned to 2% O_2_. Although cleavage rates remained unaffected across conditions, significant differences were observed in blastocyst formation rates. Under low O_2_, 36% of embryos progressed to the blastocyst stage, compared to just 13% in atmospheric conditions. In contrast, embryos exposed to ultra-low O_2_ had markedly reduced developmental potential, with only 4.6% reaching the blastocyst stage [102].

The studies discussed above provided substantial evidence supporting the use of O_2_ tension <20% for optimal embryo development. Although the precise optimal O_2_ tension varies among species, the majority of research indicates that culturing embryos using 5% O_2_ is beneficial. However, to date, studies investigating the effects of low O_2_ (5%) and ultra-low O_2_ (1–2%) tension during in vitro embryo culture have produced conflicting results, warranting further research. A potential reason for the limited influence of O_2_ tension on pregnancy outcomes may be that Day-3 embryos, which lack differentiated trophectoderm cells at the cleavage stage, have reduced sensitivity to hypoxic conditions. Another contributing factor could be the limited statistical power of some studies. However, based on meta-analyses, findings generally support 5% O_2_ for embryo culture (Table 2).

## 4. Oxygen Uptake and Metabolism in Pre-Implantation Embryos

**Figure 2:**
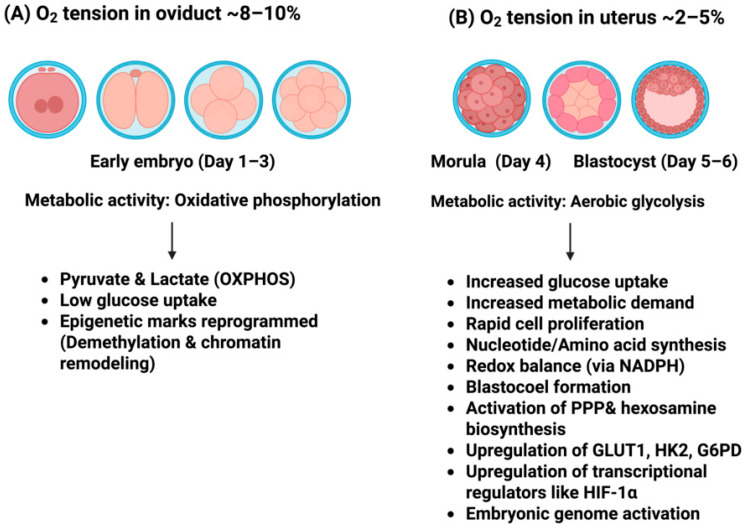
Oxygen gradient and metabolic reprogramming in pre-implantation embryos. (**A**) In the oviduct, where oxygen tension is relatively higher (~8–10%), early embryos rely predominantly on oxidative phosphorylation for ATP production, maintaining a metabolically quiescent state and balanced redox homeostasis. (**B**) In the uterine cavity, oxygen tension is lower (~2–5%), promoting a shift toward aerobic glycolysis (the Warburg effect) [80]. This metabolic adaptation, mediated by HIF-1α activation, enhances glucose uptake and lactate production, supporting rapid cell proliferation, blastocoel formation, and preparation for implantation. NADPH: nicotinamide adenine dinucleotide phosphate, PPP: pentose phosphate pathway, G6PD: Glucose-6-phosphate dehydrogenase, GLUT1: Glucose Transporter 1, HK2: Hexokinase 2, HIF-1α: Hypoxia-inducible factor 1-alpha. Created in BioRender. Mehdinejadiani, SH. (2026) https://BioRender.com/7qm2flx.

Reduced O_2_ tension within the oviduct and uterus is considered a key physiological factor in modulating the developmental competence of embryos [18]. As embryos progress from cleavage to the blastocyst stage, O_2_ consumption increases, and its interaction with the embryo, both in vivo and in vitro, can influence metabolic activity, ROS production, and apoptosis [115]. During the pre-implantation phase, mammalian embryos primarily depend on oxidative metabolism through the Krebs cycle and oxidative phosphorylation. However, as development progresses to the blastocyst stage, there is increased reliance on the Embden–Meyerhof pathway (glycolysis; Figure 2) [115,116]. The shift from oxidative phosphorylation to aerobic glycolysis is tightly regulated and may be susceptible to disruption by in vitro culture conditions. Such stressors may accelerate this metabolic switch, leading to premature glucose utilization, which may adversely affect embryonic development [117]. This transition reflects an embryo’s progression from a metabolically quiescent state to a more active metabolic profile, supporting heightened energy demands associated with implantation [116].

Analyses of metabolic profiles of high-quality Day-3 embryos indicated that concentrations of key metabolites, e.g., glucose and fatty acids, are comparable between atmospheric and low-O_2_ conditions [26,86]. However, more recent research on human blastocysts reveals that changes in O_2_ tension (e.g., from 5 to 2%) can significantly influence the abundance of anabolic amino acids and metabolites related to redox balance. These findings, including the upregulation of Mucin 1, highlighted the role of O_2_ in modulating embryo metabolism, particularly during the later stages of pre-implantation development [105].

Kelley et al. reported that mouse embryos cultured under atmospheric O_2_ conditions had decreased uptake of glucose and aspartate, with increased production of glutamate and ornithine, when compared to embryos cultured under 5% O_2_ [77]. In mouse embryos cultured under low-O_2_ conditions, there was increased pyruvate oxidation and formation of blastocysts with more cells and fewer apoptotic cells [2]. Similarly, reducing O_2_ tension from atmospheric to low tension (5%) enhanced catabolic utilization of glucose in pre-implantation ovine embryos [118].

Disruptions in metabolic regulation can also influence apoptotic pathways, with the impact potentially exacerbated by DNA damage resulting from ROS. In contrast, embryos with quiet metabolism are typically associated with more balanced apoptotic regulation [109]. In porcine embryos, exposure to low O_2_ (5%) tension decreased H_2_O_2_ production and reduced DNA fragmentation, compared to embryos cultured under atmospheric O_2_ conditions [12]. A comparative proteomic analysis of hatched blastocysts cultured under 5% versus 20% O_2_ conditions identified 43 differentially expressed proteins (DEPs), primarily involved in glycolysis, fatty acid degradation, inositol phosphate metabolism, and terpenoid backbone biosynthesis. Therefore, buffalo embryos cultured under 5% O_2_ tension may have enhanced developmental potential, potentially due to a more pronounced Warburg effect (aerobic glycolysis). Furthermore, based on proteomic profile, increased lipid catabolism, higher cholesterol and an elevated unsaturated-to-saturated fatty acid ratio may underlie the improved cryo-survival of embryos cultured at low O_2_ tension [80].

Embryos produced in vitro may be affected by shifts in substrate oxidation patterns, elevated glycolysis, diminished mitochondrial respiration, or enhanced aerobic lactate production, with conversion of glucose to lactate [117]. Elevated O_2_ tension may impair glucose oxidation, potentially representing an adaptive response by the embryo to non-physiological culture environments. Disruptions in cellular metabolism induced by suboptimal culture conditions can impact cell cycle regulation, cytoskeletal dynamics, membrane integrity, and calcium signaling. Moreover, glycolysis may be prematurely activated in response to environmental stress or compromised embryo quality [117]. Boskovic et al. reported that atmospheric O_2_ conditions interfered with the expression of genes critical for energy metabolism in bovine blastocysts. Although glucose was expected to be the primary energy substrate, genes related to glycolysis, gluconeogenesis, and amino acid synthesis were not upregulated in blastocysts cultured under 20% O_2_ [102]. This metabolic insufficiency may explain reduced developmental competence, including decreased mitochondrial DNA (mtDNA) content observed in atmospheric O_2_-exposed mouse blastocysts [89]. This issue gains further importance in light of recent evidence suggesting that glucose gradients affect gastrulation and mesoderm formation [119,120].

Although embryos have a degree of plasticity to O_2_ variations, minimizing environmental stress is essential to avoid diverting energy toward stress responses, which may compromise viability, implantation potential, and pregnancy outcomes [1,101,121]. In cultured embryos, oxidative stress is frequently associated with cytoplasmic fragmentation, a marker of compromised embryo quality. Importantly, even without overt morphological abnormalities such as fragmentation or developmental arrest, oxidative damage may impair developmental competence of the embryo in humans [122]. Belli et al. reported that culturing mouse embryos in vitro under either low or atmospheric O_2_ conditions altered mitochondrial morphology, including vacuolated mitochondria, cytoplasmic vacuolization, and the formation of multivesicular bodies across all stages of embryo development. Furthermore, the blastocysts had reduced heterochromatin content, disrupted membrane integrity in trophectoderm and ICM, increased residual bodies, and elevated glycogen granules within the cytoplasm. Overall, results suggested that in vitro culture, particularly at atmospheric O_2_, induces stage-dependent ultrastructural modifications in mouse pre-implantation embryos [89,90]. As mitochondria are pivotal to cellular energy metabolism, in vitro culture, especially under conditions of elevated O_2_ tension, may compromise mitochondrial structure and function, and adversely affect embryonic development [123]. Elevated O_2_ tension during in vitro culture may increase metabolic activity in pre-implantation embryos, potentially accelerating developmental progression, often associated with diminished embryonic viability. Consequently, evaluating markers of metabolic health requires assessing the developmental performance of embryos cultured under varying O_2_ tension.

## 5. Impacts of Oxygen Tension on Gene Expression in Pre-Implantation Embryos

### 5.1. Oxygen Tension Modulates Genome-Wide Differential Expression of Genes in Pre-Implantation Embryos

The effects of O_2_ tension on gene expression in embryos derived from IVF/ICSI have been variable, which is attributed to differences in culture conditions, including co-culture systems and media composition. Variations in O_2_ tension caused differential gene expression related to embryonic metabolism, even when developmental rates remain comparable [21,124].

Mantikou et al. investigated how O_2_ tension affects gene expression in human pre-implantation embryos. Embryos that progressed to the morula or blastocyst stage within 2 days of culture under either 5% or 20% O_2_ were analyzed using microarray hybridization to assess genome-wide expression patterns. There were 183 differentially expressed genes (DEGs) associated with O_2_ tension. Culture under low-O_2_ conditions (5%) led to the upregulation of 117 genes involved in biological processes such as cell morphogenesis and muscle contraction. In contrast, embryos cultured in high O_2_ (20%) had an upregulation of 66 genes primarily linked to sensory perception and stimulus response [125]. While these transcriptomic changes provide insight into potential biological pathways influenced by oxygen tension, their relationship with clinically relevant outcomes such as implantation, euploidy, or live birth remains incompletely defined.

In 2025, Boskovic et al. investigated whether O_2_ tension influences embryonic genome activation (EGA) in bovine embryos. They reported that embryos cultured under either low- or atmospheric O_2_ conditions were capable of initiating EGA by the 16-cell stage; however, atmospheric O_2_ tension delayed the degradation of maternal transcripts during EGA, a process essential for reprogramming embryonic cell lineages. Subsequent transcriptomic analyses revealed substantial differences in gene expression profiles between the two conditions. Specifically, 253 genes were upregulated under low O_2_ and 260 under atmospheric O_2_, with 167 genes commonly upregulated across both conditions. Notably, 86 genes were uniquely upregulated in low O_2_ and 93 in atmospheric O_2_. Based on gene ontology (GO) analysis, upregulated genes under low-O_2_ conditions were primarily associated with molecular binding, translational activity, RNA biosynthesis, and RNA metabolism. These functional categories implied that hypoxic embryos were undergoing preparations for new transcriptional events, DNA replication, and subsequent developmental transitions. Although similar functional themes were observed in atmospheric O_2_, more genes were involved in these processes under low O_2_ [102]. However, as these findings are derived from bovine models, extrapolation to human IVF outcomes should be made cautiously and requires confirmation in human studies.

Bovine blastocysts cultured under ultra-low-O_2_ conditions (sequential culture 6 to 2%) exhibited a higher number of downregulated genes relative to the 16-cell stage compared to blastocysts developed under low-O_2_ (monophasic 6%) conditions. Despite this, energy metabolism profiles (reflected by upregulated DEGs) were comparable between embryos cultured under ultra-low O_2_ and low O_2_. In contrast to blastocysts cultured under atmospheric O_2_ conditions, those exposed to ultra-low O_2_ exhibited upregulation of genes involved in glycolysis, amino acid synthesis, and fatty acid biosynthesis [102]. In a comparable study with a limited sample size, a transcriptomic analysis of human blastocysts revealed that the biphasic O_2_ strategy (5 to 2%) modulated key pathways associated with embryonic development and implantation competence, including cellular assembly and organization, DNA replication, recombination and repair, cell cycle regulation, cell survival and death, organismal functions, organogenesis, and embryonic morphological development. Furthermore, this approach influenced the expression of genes that support DNA repair and cell proliferation, and maintain embryonic stem cell pluripotency, while concurrently downregulating genes involved in apoptosis and embryonic cell death [106].

Conversely, Morin et al. observed no significant differences in transcriptional profiles of human blastocysts cultured under a constant 5% O_2_ environment compared to those cultured under a sequential 5 to 2% O_2_ [100]. Moreover, the expression of genes associated with trophectoderm development and placentation was evaluated in human embryos cultured under 2, 5, or 20% O_2_ conditions. There were no significant differences between the 2 and 5% O_2_ groups; however, there were notable differences for the 20% O_2_ group compared to other groups [33]. These results suggest potential biological effects, but further well-powered human studies are required to confirm clinical relevance.

### 5.2. Oxygen Tension Modulates Expression of Genes Involved in Regulation of Oxidative Stress and Embryo Metabolism

Oxidative homeostasis is widely recognized as a key factor influencing the quality of embryos produced in vitro. Unlike the in vivo environment, in vitro culture deprives embryos of both enzymatic and non-enzymatic antioxidants present in the female reproductive tract [126]. Consequently, culturing embryos under atmospheric O_2_ tension can lead to excessive ROS, triggering oxidative stress in the absence of adequate antioxidant defenses.

A central player in the cellular response to oxidative stress is the NFE2L2 (NRF2) signaling pathway. NRF2 activates the transcription of antioxidant genes such as Manganese superoxide dismutase (MnSOD), peroxiredoxin (PRDX), and Superoxide dismutase (SOD)1 by binding to antioxidant response elements (AREs) in their promoters [127]. Under normal conditions, KEAP1 retains NRF2 in the cytoplasm, targeting it for degradation. However, under oxidative stress, NRF2 dissociates from KEAP1, translocates to the nucleus, and initiates the antioxidant response.

Interestingly, embryos cultured in 20% O_2_ had an elevated expression of NRF2, consistent with increased oxidative stress, whereas KEAP1 expression was higher in embryos maintained at 5% O_2_, possibly reflecting reduced activation of the pathway under more physiological conditions [127]. Embryos cultured under 20% O_2_ had an elevated expression of classical antioxidant genes such as SOD1, SOD2, PRDX1, CAT, GPX1, and ARO, likely to counteract increased oxidative stress [127,128,129].

The upregulation of NRF2 expression was reported in mouse embryos cultured under 2% O_2_ compared to in vivo-derived blastocysts [92]; this appeared to reflect a stress response mechanism triggered by exposure to a non-physiological hypoxic environment. This response is comparable to the elevated NRF2 expression in bovine embryos cultured under 20% O_2_ conditions [127], suggesting that deviations from physiological O_2_ tension in either direction can elicit oxidative stress signaling pathways. Given that the transcription factor NRF2 activates the promoter region of heat shock factor 1, this interaction upregulates inducible HSP70 expression as part of the cellular response to oxidative stress [130], whereas mRNA expression was elevated in embryos derived in vitro compared to those developed in vivo [95]. The culture of mouse embryos under 2% O_2_ markedly elevated the expression of HSP70, a 7.55-fold increase compared to embryos maintained at 5% O_2_ [92]. The elevated HSP70 expression in human embryos cultured under 2% O_2_ (Days 1–5) may reflect a cellular stress response to significantly increased Caspase-3 expression [110]. Moreover, increased HSP70 expression was also reported in human embryos cultured under 20% O_2_ [33].

Elevated O_2_ tension during in vitro embryo culture increased ROS in cytoplasm [4,8,11,12], which may trigger apoptotic pathways and alter transcriptional profiles of stress-responsive genes. Bcl-2, a key anti-apoptotic protein in the NRF2-regulated oxidative stress response pathway, had significantly elevated expression in mouse blastocysts subjected to oxidative stress induced by H_2_O_2_ [131]. Moreover, COCs and blastocysts cultured under 5% O_2_ had an elevated expression of the anti-apoptotic marker Bcl-2, alongside a reduction in the pro-apoptotic factor BAX compared to other groups in a yak model [61]. Similarly, human embryos cultured under 20% O_2_ had significantly elevated expression levels of stress- and apoptosis-related genes, including BAX, Glucose-6-phosphate dehydrogenase (G6PD), MnSOD, and HSP70, when compared to those cultured under reduced O_2_ conditions (2 or 5% O_2_); therefore, high-O_2_ tension may induce cellular stress responses impacting embryo quality [33].

PRDX1, a major H_2_O_2_ scavenger in early embryos, is transcriptionally regulated by NRF2. Proteomic analyses demonstrated that PRDX1 is abundantly expressed in the pronuclei of zygotes, acting as a key antioxidant during epigenetic reprogramming [132]. Though less studied in embryos, PRDX5 (a mitochondrial isoform) enhances blastocyst development by protecting cells against oxidative stress through the regulation of ROS and reactive nitrogen species (RNS) [133,134]. G6PD is a key enzyme in the pentose phosphate pathway, responsible for generating NADPH, which is essential for cellular antioxidant response. Similar to MnSOD, G6PD enhances the antioxidant capacity of blastocysts, particularly with elevated ROS, e.g., culture at 20% O_2_. Notably, G6PD expression levels are higher in bovine embryos cultured in vitro compared to those developed in vivo and are further elevated in embryos exposed to higher O_2_ tension [95].

The downregulation of the HIF-1 signaling pathway, a key component of the cellular response to hypoxia, has been observed [102]. HIF primarily mediates a hypoxic transcriptional response, activating the expression of genes involved in glucose uptake, glycolysis, angiogenesis, cell proliferation, autophagy, erythropoiesis, and hematopoiesis [21,135,136,137]. Ma et al. [93] reported that low O_2_ tension caused a slight increase in HIF-1α mRNA expression; furthermore, HIF-2α expression was elevated and primarily localized within the nucleus in mouse blastocysts cultured under 3% O_2_ versus those cultured at 20% O_2_.

Sharing 48% amino acid sequence identity with HIF-1α, HIF-2α has similar functional domains and is capable of activating genes containing a hypoxia response element. In mouse embryos and blastocysts, a culture under 3% O_2_ resulted in increased transcription of MnSOD and PRDX5 compared to those cultured under 20% O_2_ [93]. Perhaps reduced O_2_ conditions strengthen the antioxidant defense system by neutralizing extracellular ROS, fostering a low-oxidative stress environment conducive to embryo development [93]. Moreover, low-O_2_ conditions in embryos upregulate the production of antioxidant enzymes, thereby mitigating ROS during later developmental stages [93,95]. Knockout animal studies identified MnSOD as a downstream target of HIF-2α, regulating ROS balance and preserving mitochondrial homeostasis [138,139].

VEGF, an HIF target gene, expressed at higher levels under low O_2_ tension, has a critical role in modulating endometrial vascular permeability by directly stimulating angiogenesis, recruiting endothelial cells, and promoting their proliferation, suggesting that embryos cultured in low-O_2_ environments may have an enhanced capacity for angiogenesis during implantation. Alterations in VEGF expression were implicated in recurrent implantation failure. Moreover, concurrent upregulation of both GLUT-3 and VEGF under low-O_2_ conditions appears to support embryonic development in the early pre-implantation phase [93,140,141]. The downregulation of HIF-1 signaling in embryos cultured under atmospheric conditions may decrease developmental potential, even if these effects are not apparent during initial stages of development.

In yak, significant differences were detected in transcription levels of metabolism-related genes, antioxidant response genes, apoptosis genes, oocyte competence genes, and embryonic developmental markers in COCs and blastocysts matured with 5% O_2_ tension compared to 20, 10 or 1% O_2_ [61]. Analysis of metabolic gene and protein expression revealed that GLUT1, GAPDH, and LDHA were upregulated in COCs cultured with low O_2_ (5%) tension, whereas G6PD expression was decreased at 5% O_2_. These changes are indicative of a metabolic shift toward enhanced glucose uptake and anaerobic glycolysis, a well-recognized adaptation to low-oxygen environments. Oxygen tension also influenced genes associated with oocyte developmental competence. The poly(A) mRNA content of CCNB1, a key regulator of meiotic progression, as well as GATM and LUM, was significantly reduced at 5% O_2_, suggesting altered regulation of cell cycle control and extracellular matrix-associated signaling under low-oxygen conditions. In contrast, GREM1 poly(A) mRNA and protein levels were elevated at 5% O_2_ compared to 10 or 20% O_2_, supporting its proposed role as an oxygen-sensitive oocyte-derived factor linked to follicular signaling and developmental competence. Regarding redox regulation, the antioxidant enzyme GPX1, which contributes to cellular protection against oxidative stress by detoxifying hydrogen peroxide, was expressed at lower levels under 5% O_2_ than the other groups, whereas MnSOD expression remained unchanged [61]. Under low oxygen and limited glucose availability, ENO1, a key glycolytic enzyme, was upregulated, reflecting a metabolic shift toward glycolysis in response to hypoxic stress. This metabolic adaptation is paralleled by the activation of hypoxia-responsive pathways involved in mitochondrial regulation and cell survival. In this context, in bovine models, BNIP3, a hypoxia-inducible regulator of mitochondrial turnover and cell survival, shows increased expression under low-oxygen culture conditions and may contribute to oxygen-dependent regulation of apoptotic and adaptive responses during in vitro maturation [142].

Transcription levels of embryonic developmental markers CDX2, POU5F1, SOX2, and NANOG were elevated in IVF-derived blastocysts matured in 5% O_2_ tension [61]. Furthermore, 2% O_2_ tension modulated the expression of GLUT-1, GLUT-3, and VEGF in post-compaction mouse embryos [20]. Several studies have demonstrated that culturing embryos under low-O_2_ conditions significantly upregulated GLUT-3 expression compared to those cultured at 20% O_2_. This increased expression of GLUT-3 enhances glucose uptake, facilitating sufficient ATP production while minimizing ROS and increasing mitochondrial membrane potential, promoting embryonic development [20,29,93,143]. Glucose is a pivotal regulator of embryonic metabolic pathways, including the pentose phosphate and hexosamine biosynthetic pathways, which impact trophectoderm differentiation through post-transcriptional modulation of the Hippo signaling cascade [144,145,146]. This pathway, essential for determining cell fate and spatial organization as well as for blastocyst formation and implantation, was downregulated in bovine embryos cultured under atmospheric conditions [102]. Additionally, glucose mediates the glycosylation of the transcriptional co-activator, Yes-associated protein 1 (YAP1), which promotes the expression of key trophectoderm-specific transcription factors, e.g., CDX2 (Caudal type homeobox 2) and GATA3 (GATA binding protein 3) through the Hippo signaling cascade [144,145].

**Figure 3:**
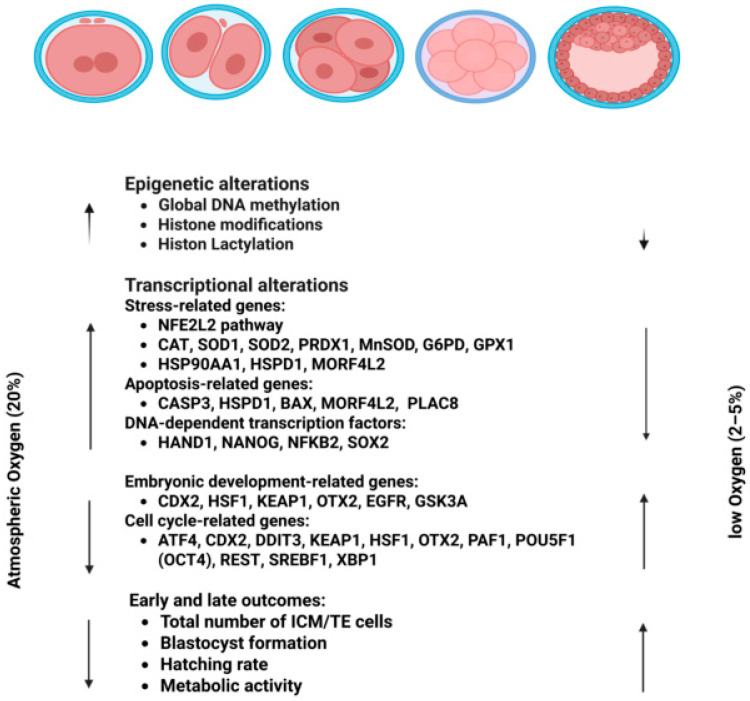
The effects of oxygen tension on transcriptional and epigenetic alterations during embryo culture. Under low oxygen levels (2–5% O_2_), genes promoting embryo development, metabolism, and cell survival are upregulated, whereas high oxygen (20% O_2_) induces expression of stress-related genes and may compromise developmental potential. Atmospheric oxygen elevates global DNA methylation and histone modifications, whereas hypoxia (2–5% O_2_) reduces LDHA expression and glycolytic flux, lowering lactate concentrations and histone lactylation. Arrows indicate the direction of changes (↑ upregulated; ↓ downregulated). NFE2L2: Nuclear factor, erythroid 2 like 2 (NFE2L2/NRF2) pathway, CAT: Catalase, SOD1: Superoxide dismutase 1, SOD2 (MnSOD): Superoxide dismutase 2, mitochondrial (Manganese SOD), PRDX1: Peroxiredoxin 1, G6PD: Glucose-6-phosphate dehydrogenase, GPX1: Glutathione peroxidase 1, HSP90AA1: Heat Shock Protein 90 Alpha Family Class A Member 1, HSPD1: Heat Shock Protein Family D Member 1 (Hsp60), MORF4L2: Mortality Factor 4 Like 2, CASP3: Caspase 3, BAX: BCL2-associated X protein, PLAC8: Placenta-specific 8, HAND1: Heart and Neural Crest Derivatives Expressed 1, NANOG: Nanog Homeobox, NFKB2: Nuclear Factor Kappa B Subunit 2, SOX2: SRY-Box Transcription Factor 2, CDX2: Caudal Type Homeobox 2, HSF1: Heat Shock Factor 1, KEAP1: Kelch Like ECH-Associated Protein 1, OTX2: Orthodenticle Homeobox 2, EGFR: Epidermal Growth Factor Receptor, GSK3A: Glycogen Synthase Kinase 3 Alpha, ATF4: Activating Transcription Factor 4, DDIT3: DNA Damage Inducible Transcript 3, PAF1: RNA Polymerase II Associated Factor 1, POU5F1: POU Class 5 Homeobox 1, REST: RE1-Silencing Transcription Factor, SREBF1: Sterol Regulatory Element Binding Transcription Factor 1, XBP1: X-Box Binding Protein 1. Created in BioRender. Mehdinejadiani, SH. (2026) https://BioRender.com/7qm2flx.

Collectively, these findings suggested that in vitro embryo culture under atmospheric O_2_ tension disrupts the expression of critical transcription factors regulating diverse cellular pathways, reducing embryo development, quality, and viability, primarily due to increased ROS (Figure 3). However, these observations from animal studies should not be assumed to predict human embryo responses without further validation.

## 6. Impacts of Oxygen Tension on Epigenetic Programming in Early Embryos and Extraembryonic Tissues

### 6.1. Epigenetic Regulators Affected by Oxygen Tension During Pre-Implantation Development

Limited information is available regarding the impacts of varying O_2_ tension on regulatory mechanisms, particularly epigenetic modifications. Effective epigenetic regulation during pre-implantation development is essential for appropriate gene expression patterns enabling embryo growth and viability [147,148,149]. Moreover, epigenetic mechanisms seem to be particularly sensitive to variations in culture conditions [150,151]. Oxygen is a key determinant in early embryogenesis, influencing both epigenetic modifications and metabolic pathways essential for proper development (Figure 3) [152,153]. During pre-implantation development, epigenetic mechanisms, including DNA methylation and histone modifications, have critical roles in regulating both imprinted and non-imprinted genes, initiating X chromosome inactivation, and modulating telomere length [154]. Recent findings linking O_2_ tension to transcriptional regulation of epigenetic modifiers provide supporting evidence for the role of O_2_ tension in the establishment of genomic imprints [155,156,157]. Histone modification is a crucial epigenetic mechanism in embryo development [158,159,160]. During pre-implantation development, histone modifications have two distinct patterns: a stable, persistent mark and a dynamic, reversible mark, with essential roles in regulating gene expression and embryonic progression [161].

Increased global DNA methylation was observed in bovine pre-implantation embryos exposed to 20% O_2_ tension, suggesting oxidative stress may influence alterations in the embryonic epigenome [23]. Gaspar et al. examined the impacts of O_2_ tensions (5 and 20%) during in vitro bovine embryo production on epigenetic remodeling, focusing on the repressive mark H3K9me2 (a repressive histone modification linked to transcriptional inhibition and heterochromatin formation) and the permissive mark H3K4me2 (a permissive mark indicative of active or transcriptionally competent chromatin), as well as the expression of remodeling enzymes. In that study, O_2_ tension significantly influenced both histone modifications, with embryos cultured under atmospheric O_2_ tension exhibiting increased levels of both repressive and permissive marks. However, no significant differences were observed in the expression of remodeling enzymes in morulae or blastocysts in response to O_2_ tension variations [24].

The impact of O_2_ tension has been examined to determine its role in regulating the transcription of key enzymes involved in chromatin remodeling. In mouse embryonic stem cells, O_2_ tension influenced the expression of the Ten-Eleven-Translocation (TET) enzyme family and the de novo DNA methyltransferase *Dnmt3a*. These transcriptional shifts were linked to altered regulation of several imprinted genes, including *H19*, *Igf2*, *Igf2r*, and *Peg3*. Similarly, bovine embryos generated in vitro and exposed to atmospheric O_2_ tension had disrupted expression of *TET1*, *TET3*, *DNMT3a*, and the methylation co-factor *HELLS*. Atmospheric O_2_ exposure also affected transcript levels of components of the Polycomb repressive complex (*EED*, *EZH2*), the histone methyltransferase *SETDB1*, and several histone demethylases (*KDM1A*, *KDM4B*, *KDM4C*). These molecular alterations were accompanied by diminished embryo viability and reduced expression of pluripotency markers *NANOG* and *SOX2*. Collectively, these findings emphasized oxygen’s regulatory capacity over transcriptional networks controlling chromatin-modifying enzymes, with downstream effects on both pluripotency maintenance and genomic imprinting [155].

Lactylation has recently emerged as a novel epigenetic modification primarily, associated with histone proteins and implicated in the regulation of diverse cellular processes; however, its specific function during early embryonic development is largely unexplored [162]. As lactate concentrations peak at the blastocyst stage [163], and histone lactylation is directly influenced by lactate [162], the elevated histone lactylation observed at this stage may, at least in part, be attributed to the high intracellular lactate concentration in blastocyst-stage embryos. Yang et al. investigated associations among O_2_ tension, LDHA activity, and histone lactylation in pre-implantation embryos; they reported that the inhibition of Lactate Dehydrogenase A (LDHA) activity with GSK2837808A reduced histone lactylation and decreased developmental rates of pre-implantation embryos under atmospheric O_2_, confirming that lactate generation via LDHA is essential for maintaining normal histone lactylation and embryonic progression. Oxygen tension influences this process indirectly by modulating LDHA expression and activity: Under gradient O_2_ tensions (5 to 2%), embryos had reduced LDHA expression and lower glycolytic flux, resulting in diminished lactate production and consequently decreased histone lactylation marks such as H3K18la and H3K23la. These epigenetic changes may disrupt gene expression programs critical for early development [164]. Notably, Yang et al. [164] also reported that ultra-low O_2_ (2%) conditions did not significantly alter histone acetylation (H3K23ac and H3K18ac), indicating a functional distinction between histone acetylation and lactylation during pre-implantation development.

Collectively, these findings indicate that ultra-low O_2_ tension reduces LDHA expression and lactate production, thereby diminishing histone lactylation and potentially influencing epigenetic regulatory mechanisms in pre-implantation embryos [164]. Notably, similar alterations in metabolic flux and epigenetic markers have also been reported under low-O_2_ conditions (5%), where mouse blastocysts exhibit increased glucose oxidation and reduced glycolytic activity due to LDHA downregulation, resulting in decreased lactate production and histone lactylation [29].

### 6.2. Long-Term Epigenetic Alterations for Placenta and Postnatal Health

Epigenetic reprogramming during pre-implantation development is highly sensitive to the environment of surrounding gametes and early embryos. Oxygen tension during in vitro embryo culture has emerged as a critical determinant of epigenetic stability. Evidence from both human and animal studies suggests that variations in O_2_ tension can influence DNA methylation patterns in embryonic and placental tissues, potentially affecting fetal development and postnatal outcomes. A murine study comparing IVF under two O_2_ tensions (5 and 20%) with natural conception demonstrated an increased frequency of random epigenetic alterations at imprinted genes in placental tissue following IVF; DNA methylation patterns at imprinted loci in the placenta were influenced by O_2_ tension. Specifically, placentas derived from embryos cultured under 20% O_2_ exhibited significantly altered methylation at the Peg3 imprinting control region (ICR) compared to placentas from naturally conceived embryos. However, at the H19/Igf2 ICR, aberrant methylation was observed in both IVF groups, regardless of O_2_ tension during culture. Notably, as the frequency of these epigenetic errors did not differ between the two O_2_ conditions, embryonic tissue appeared to be protected from methylation alterations [165]. In a study on human placental tissue to evaluate global DNA methylation at repetitive elements (specifically LINE1 sequences) in placentas from naturally conceived pregnancies compared to those conceived via IVF, placentas from embryos cultured under atmospheric O_2_ conditions (20%) had significant alterations in LINE1 methylation relative to those from natural conceptions. In contrast, placentas from embryos cultured under low O_2_ tension (5% O_2_) demonstrated methylation patterns more closely resembling those of in vivo conceptions. Perhaps exposure to atmospheric O_2_ tension during early embryonic development may exert persistent epigenetic effects in offspring [25].

O_2_ tension during IVM remains contentious, with studies reporting both beneficial [47,166] and detrimental [59,60] effects. Inconsistencies among findings may be attributed to variations in maturation media composition, particularly glucose concentration [167]. Transcriptomic analysis of oocytes cultured under atmospheric conditions demonstrated chromatin reorganization and an upregulation of genes associated with female reproductive function. Conversely, oocytes cultured under 5% O_2_ exhibited a transcriptional shift toward cellular stress response pathways. Naillat et al. established a functional assay to assess oocyte methylation by utilizing inhibitors that suppress both methylation and transcription. Their findings revealed that reducing histone methylation and transcription decreased DNA methylation levels at CpG islands (CGIs) within gene bodies of grown oocytes (transcribed regions of genes). These findings underscore distinct variations in DNA methylation and transcripts between oocytes cultured under various O_2_ tensions in vitro and those matured in vivo. Oocytes exposed to 20% O_2_ had a stronger correlation with in vivo-matured murine oocytes in terms of DNA methylation and transcription, suggesting that 20% O_2_ tension better supported oocyte maturation in ex vivo culture conditions (Figure 1) [56]. Moreover, global DNA methylation pattern in the maternal pronucleus of bovine zygotes is influenced by oocyte maturation under low O_2_ tension, which may affect early embryonic development [58]. These findings indicated that adequate (higher) O_2_ availability supports maternal DNA methylation, whereas reduced O_2_ tension may impair it. Furthermore, methylation patterns established in the oocyte have a crucial regulatory role in murine trophoblast development, influencing gene expression essential for placental formation and function [168]. Overall, findings from animal studies indicate that oxygen levels play a role in regulating epigenetic and transcriptional processes during early development. However, their relevance to human pre-implantation embryos remains to be fully established, and extrapolation to human IVF outcomes should therefore be interpreted with caution.

Although human clinical data regarding postnatal consequences of varying O_2_ tensions during embryo culture remain limited, existing studies generally report no significant differences in immediate neonatal outcomes such as single and twin delivery rates, rates of preterm, birthweight, gestational age, or congenital anomalies [83,84]. However, a large retrospective human cohort study published in 2023, analyzing 31,566 IVF cycles, revealed that embryos cultured in 5% O_2_ had improved embryo quality and cumulative live birth rates compared to those cultured in 20% O_2_, and a modest (albeit statistically significant) increase in birthweight was reported in the 20% O_2_ group [85]. It is important to acknowledge that the absence of long-term follow-up in these studies precludes definitive conclusions about potential latent effects. Evidence from animal models, particularly mice, has demonstrated that embryos exposed to non-physiological O_2_ tensions, especially ultra-low tension such as 2% O_2_, can have developmental disturbances. Feil et al. reported increased rates of fetal resorption and reduced fetal weights at Day 18 in mouse embryos cultured under 2% O_2_ following compaction, despite no changes in placental morphology [94]. Collectively, these data underscored the nuanced and potentially long-term implications of in vitro O_2_ tension, reinforcing the need for further longitudinal studies to clarify its role in offspring health and disease susceptibility. Collectively, these data underscored the nuanced and potentially long-term implications of in vitro O_2_ tension, reinforcing the need for further longitudinal studies to clarify its role in offspring health and disease susceptibility.

## 7. Future Perspectives

Future research should focus on elucidating the molecular mechanisms by which O_2_ tension regulates key epigenetic modifiers such as DNA methyltransferases, histone-modifying enzymes, and non-coding RNAs. Particular emphasis should be placed on understanding the timing and reversibility of these epigenetic alterations and long-term implications for offspring health. Importantly, rather than static O_2_ tension, dynamic fluctuations more accurately mimic in vivo conditions. Investigating how O_2_ variability influences gene regulatory networks could provide critical insights into embryonic development. To achieve these aims, the development of advanced embryo culture systems capable of real-time O_2_ monitoring and modulation is essential.

To replicate the physiological O_2_ environment within culture systems, it is crucial to implement integrated mechanisms for the precise detection and regulation of O_2_ tension. Biosensors [169,170] could enable precise, localized measurements of O_2_ tension within culture droplets, allowing dynamic adjustments tailored to the embryo’s developmental stage. Furthermore, advances in microfluidic technology offer an opportunity to replicate spatial and temporal O_2_ gradients of the female reproductive tract [171,172,173]. These systems provide meticulous control over environmental conditions, perhaps enhancing developmental competence with a more physiologically appropriate environment.

Future studies should also determine whether replicating measured in vivo oxygen levels is truly optimal for in vitro development. In the absence of maternal buffering systems and physiological gradients, the functionally optimal O_2_ concentration in culture may differ from in vivo values. Establishing this in vitro-specific optimum will require a systematic evaluation of developmental competence, genomic integrity, epigenetic stability, and long-term safety across both static and dynamic oxygen strategies. Integrative comparisons of in vivo and in vitro datasets—particularly through genome and epigenome profiling—will be essential to clarify how O_2_ influences chromosomal stability, imprinting, DNA repair, and self-correction mechanisms underlying euploid blastocyst formation.

In parallel, further research should examine interactions between O_2_ tension and culture media composition. Proteomic and metabolomic analyses may identify how specific components, including amino acids, growth factors, and metabolic substrates, respond to varying O_2_ conditions and influence embryonic outcomes. The role of antioxidants also warrants careful optimization, as their efficacy likely depends on specific O_2_ contexts [174]. Finally, the integration of artificial intelligence into ART holds significant promise [175,176,177]. AI systems can synthesize complex datasets, including O_2_ dynamics, metabolic profiles, gene expression, and morphological features, to create individualized, adaptive culture protocols, perhaps improving implantation rates and clinical outcomes through personalized embryo care.

Ultimately, optimizing in vitro embryo culture systems demands a multidisciplinary effort involving molecular biology, bioengineering, systems biology, and computational intelligence. Through an integrative strategy, future innovations will improve embryo viability and developmental outcomes, ensuring the safety and viability of ART-derived offspring.

## 8. Conclusions

This review explored the impacts of O_2_ tension on in vitro embryo development, focusing on the developmental competence of embryos, metabolism, gene expression and epigenetic alterations. Accumulating evidence indicates that low O_2_ tension (closer to physiological) better supports embryo viability, quality, and developmental competence. O_2_ tension influences not only cellular metabolism but also gene regulation and epigenetic programming, both of which are critical for embryo competence and long-term health outcomes.

Culture under atmospheric O_2_ (~20%) has been associated with increased oxidative stress, leading to abnormal DNA methylation, disrupted gene networks, and impaired development. In contrast, lower O_2_ (5 to 6%) is more aligned with in vivo conditions and is supported by multiple clinical studies and randomized trials demonstrating improved embryo culture outcomes compared with atmospheric O_2_. Although O_2_ requirements vary by species, most human studies comparing 5 versus 20% O_2_ reported no advantage of atmospheric conditions. Taken together, currently available outcome-based human evidence broadly supports the use of approximately 5% O_2_ as the preferred clinical culture condition.

Evidence supporting ultra-low O_2_ (e.g., 2%) remains limited, as it is largely derived from experimental, animal, or molecular-level investigations rather than well-powered clinical outcome studies. Similarly, a biphasic O_2_ strategy (5% during the first 3 days, followed by 2%) represents a biologically plausible and potentially beneficial approach; however, at present, it should be considered hypothesis-generating and requires validation in well-designed prospective human clinical trials before routine clinical implementation.

Although embryos exhibit a degree of plasticity to O_2_ variations, minimizing environmental stress is essential to avoid diverting energy toward stress responses, which may compromise viability, implantation potential, and pregnancy outcomes. Lower O_2_ tension has facilitated improved blastocyst formation and supports elective single embryo transfer (eSET). However, future research should prioritize correlating molecular and epigenetic findings with clinically relevant reproductive outcomes, including gene expression and DNA methylation, particularly of imprinted and non-imprinted genes in blastocysts, fetal tissues, and placenta. As O_2_ tension can influence not only early embryonic development but also long-term and transgenerational health outcomes, gaining a more comprehensive understanding of these underlying mechanisms is essential for refining in vitro culture conditions and enhancing the safety and effectiveness of ART.

## Figures and Tables

**Table 1:** Effect of oxygen tension on in vitro maturation (IVM).

Species/Ref.	Oxygen Tension(s) Tested During IVM (% O_2_)	Optimal Oxygen Tension for IVM	Key IVM Outcomes	Overall Conclusion
Mouse [54,55,56]	2, 5, 10, 20	No single optimum	IVM O_2_ did not affect maturation or fertilization but modified epigenetic programming, blastocyst cell allocation, apoptosis, and fetal/placental outcomes.	Oxygen tension during IVM influences oocyte quality and developmental programming rather than nuclear maturation per se.
Porcine [52,53,57]	5, 20 (CAPA-IVM)	5%	Nuclear maturation largely unaffected by O_2_; 5% O_2_ enhanced oocyte activation potential and promoted glycolytic gene expression in cumulus cells.	Low O_2_ improves oocyte competence and metabolic programming without altering maturation rates.
Bovine [46,47,58,59,60]	5, 10, 20	20%	Reduced O_2_ markedly decreased MII rates and fertilization and increased polyspermy; under specific conditions, low O_2_ altered metabolism and epigenetic programming.	Atmospheric O_2_ generally supports optimal bovine IVM, while reduced O_2_ may impair maturation and fertilization despite metabolic adaptations.
Buffalo [50,51]	5, 20	5%	5% O_2_ increased MII rates, cumulus expansion, glycolytic activity, and reduced oxidative stress; antioxidants partially rescued high-O_2_ effects.	Reduced O_2_ supports meiotic maturation and metabolic competence in buffalo oocytes.
Yak [61]	1, 5, 10, 20	5%	5% O_2_ yielded highest maturation rates, optimal gene expression profiles, and lowest apoptosis; 1% O_2_ was detrimental.	Yak oocyte maturation is highly oxygen-sensitive, with 5% O_2_ providing optimal conditions.
Human [62,63]	5, 20 (CAPA-IVM)	Context-dependent	5% O_2_ improved metabolic efficiency during pre-IVM but reduced maturation and fertilization compared with 20% O_2_.	Oxygen effects during human IVM are phase-specific; metabolic benefits of low O_2_ may not translate into improved maturation outcomes.

**Table 2:** Studies exploring the effect of oxygen tension on embryo development.

Species	Study Design	Developmental Stage	Optimal O_2_ (%)	Key Outcomes	Conclusion
Mouse [20,27,89,90,91,92,93,94]	Monophasic	Morula/Blastocyst	2–5%	2% O_2_ ↑ GLUT-1, GLUT-3, VEGF; 3–5% O_2_ ↑ blastocyst and hatch rates, lower ROS, better mitochondrial structure	Low O_2_ improves pre-implantation development, mitochondrial function, antioxidant defense. Development at 2% O_2_ was significantly delayed.
Mouse [20,27,94]	Biphasic	Morula/Blastocyst	2–7%	Sequential or alternating O_2_ ↑ oxygen-regulated gene expression; similar blastocyst rates	Low O_2_ enhances embryo viability and gene regulation. 2% O_2_ negatively effects fetal development in mice.
Porcine [12,53]	Monophasic	Blastocyst	5%	5% O_2_ ↑ blastocyst formation, ↓ ROS, ↓ DNA fragmentation	Low O_2_ enhances embryo developmental ability.
Bovine [24,95,96]	Monophasic	Cleavage/Blastocyst	5%	5% O_2_ ↑ morula and blastocyst survival, ↓ apoptosis, ↑ total cell number	5% O_2_ optimal for embryo culture, maximizing survival and quality.
Bovine [19]	Biphasic	Blastocyst	2–7%	Alternating O_2_ ↑ GLUT1, ICM proportions, gene expression	Biphasic O_2_ affects post-compaction embryo gene regulation.
Buffalo [50,51,80]	Monophasic	Cleavage/Blastocyst	5%	5% O_2_ ↑ blastocyst formation, ↑ cell number, ↑ glycolysis, ↑ cryo-survival	5% O_2_ supports optimal embryo development.
Yak [61]	Monophasic	Cleavage/Blastocyst	5%	Blastocyst rates ↑, ICM/TE cell number ↑, apoptosis ↓; ultra-low 1% O_2_ ↓ cleavage and blastocyst	5% O_2_ optimizes development and blastocyst quality; 1% O_2_ severely impairs embryos.
Human embryos [17,69,71,74,81,82,84,97]	Monophasic	Cleavage	5–6%	5% O_2_ ↑ Day 3 embryo score, ↑ top-quality embryos, ↑ implantation and pregnancy rates	5% O_2_ improves pre-implantation embryo quality and cumulative live birth.
Human embryos [30,31,33,74,82,85,98,99]	Monophasic	Blastocyst	5%	↑ blastocyst formation, ↑ birth rate, ↓ apoptosis	5% O_2_ yields better blastocyst outcomes and higher live birth.
Human embryo [33,34,100,101,102,103,104,105,106]	Biphasic	Blastocyst	5→2%	Sequential culture ↑ total and usable blastocyst rates, cumulative live birth; improved metabolism and transcriptome profiles. Biphasic O_2_ promoted glycolysis and lipid metabolism, mimicking in vivo conditions.	Sequential 5→2% O_2_ enhances blastulation, embryo quality, and live birth rates.

The arrows (↑) and (↓) indicate an increase and decrease in the measured parameters, respectively.

## Data Availability

No new data were created or analyzed in this study. Data sharing is not applicable to this article.
